# Draft genome data of *Prunus avium* cv ‘Stella’

**DOI:** 10.1016/j.dib.2022.108611

**Published:** 2022-09-17

**Authors:** Richard M. Sharpe, Benjamin Killian, Tyson Koepke, Rishikesh Ghogare, Nnadozie Oraguzie, Matthew Whiting, Lee A. Meisel, Herman Silva, Amit Dhingra

**Affiliations:** a Department of Horticulture, Washington State University, Pullman WA 99164, United States; b Department of Agriculture, African Christian University, Lusaka H985+XQ3, Zambia; c Irrigated Agriculture Research and Extension Center, Washington State University, Prosser, WA 99350, United States; d Instituto de Nutrición y Tecnología de Los Alimentos (INTA), Universidad de Chile, El Libano 5524, 7830490, Macul, Santiago, Chile; e Facultad de Ciencias Agronómicas, Laboratorio de Genómica Funcional & Bioinformática, Universidad de Chile, Av. Santa Rosa 11315, 8820808, La Pintana, Santiago, Chile; f Department of Horticultural Sciences, Texas A&M University, College Station, Texas 77843, United States

**Keywords:** Genome, *Prunus avium*, High-throughput sequencing, Rosaceae

## Abstract

*Prunus avium* cv. ‘Stella’ total cellular DNA was isolated from emerging leaf tissue and sequenced using Roche 454 GS FLX Titanium, and Illumina HiSeq 2000 High Throughput Sequencing (HTS) technologies. Sequence data were filtered and trimmed to retain nucleotides corresponding to Phred score 30, and assembled with CLC Genomics Workbench v.6.0.1. A total of 107,531 contigs were assembled with 185 scaffolds with a maximum length of 132,753 nucleotides and an N_50_ value of 4,601. The average depth of coverage was 135.87 nucleotides with a median depth of coverage equal to 31.50 nucleotides. The draft ‘Stella’ genome presented here covers 77.8% of the estimated 352.9Mb *P. avium* genome and is expected to facilitate genetics and genomics research focused on identifying genes and quantitative trait loci (QTL) underlying important agronomic and consumer traits.

## Specification Table

**Table:** 

Subject	Omics: Genomics
Specific subject area	Draft genome of *Prunus avium* cv ‘Stella’ using short and long-read high throughput sequencing technologies to facilitate sweet cherry genetics, genomics and breeding research
Type of data	Genomic sequence
How data were acquired	PacBio, Illumina and Roche 454 reads were assembled with CLC Genomics Workbench v.6.0.1
Data format	Raw read data – fastq format Analyzed data – fasta format
Parameters for data collection	A total of 107,531 contigs were assembled with 185 scaffolds with a maximum length of 132,753 nucleotides (nt) and an N50 value of 4,601. Average depth of coverage was 135.87nt; median depth of coverage 31.50nt. The assembly was generated from Paired-End Illumina reads.
Description of data collection	Data were collected from processing the raw reads produced via the different sequencing platforms using the CLC Genomics Workbench v. 6.0.1.
Data source location	Washington State University Pullman, WA. United States of America 46°43′52.57" N -117°10′46.63″ W
Data accessibility	Data hosted on NCBI (https://www.ncbi.nlm.nih.gov/): NCBI accession - SRR4280447, Type: Illumina HiSeq 2000 https://www.ncbi.nlm.nih.gov/sra/SRR4280447 NCBI accession - SRR4280448, Type: 454 GS FLX Titanium https://www.ncbi.nlm.nih.gov/sra/?term=SRR4280448
Related research article	**Hewitt S, Kilian B, Hari R, Koepke T, Sharpe R, Dhingra A** (2017) Evaluation of multiple approaches to identify genome-wide polymorphisms in closely related genotypes of sweet cherry (*Prunus avium* L.). Comput Struct Biotechnol J. doi:10.1016/j.csbj.2017.03.002

## Value of the Data

• The sequence data from the first self-fertile named sweet cherry cultivar generated via mutation breeding will be useful for genomics research.
• The genomic data from ‘Stella’ cultivar are valuable for understanding the genetic diversity of sweet cherry, since this fruit crop experienced a genetic bottleneck during its domestication.
• Plant breeders, bioinformatics scientists, genomics and genetics scientists and biotechnologists will benefit from these data.
• These data can be used in developing trait-linked molecular markers and quantitative trait loci to develop superior cultivars. The collective information will also aid in performing functional characterization of genes.

## 1 Data Description

Cultivated Sweet Cherry varieties are an outcome of the domestication of the wild cherry *Prunus avium* L., and are thought to have originated in the region between the Black and Caspian Seas [1,2]. The data from *Prunus avium* cv. ‘Stella’ was generated using two different next generation sequencing platforms. Single, 8 kb paired-end (PE), and 20 kb PE reads were generated using pyrosequencing on the 454 GS FLX instrument, and 100 bp PE reads were generated using Illumina 2000 instrument. A total of 18.38 GB of data were generated and with the genome size estimated to be 352.9 Mb, these data represent 81.68X coverage of the genome. Specific details about the data are summarized in Table 1.

**Table 1:** Amount of *Prunus avium* cv. ‘Stella’ genomic data generated using 454 and Illumina sequencing platforms.

Species	Data Type	Amount	Coverage (x)

*Prunus avium* cv ‘Stella’ (sweet cherry)	454-single	1Gb	4.44
	454-8kb paired	63.7Mb	0.28
	454-20kb paired	116.5Mb	0.52
	Illumina (100bp PE)	17.2Gb	76.44
	**Total**	**18.38Gb**	81.68

A draft genome assembly of *Prunus avium* cv ‘Stella’ was developed using CLC genomics that consisted of 107,531 assembled contigs organized into185 scaffolds with a maximum length of 132,753 nucleotides (nt) and an N_50_ value of 4,601. Average depth of coverage was 135.87nt, and median depth of coverage 31.50nt. The assembly summary report is attached as Supplementary file 1. In addition contig information in terms of consensus length, total read count and reads in pairs and average coverage is summarized in Supplementary file 2.

## 2 Experimental Design, Materials and Methods

### 2.1 Leaf material and Genomic DNA Purification

Leaf material from ‘Stella’ sweet cherry cultivar was collected from WSU IAREC, Prosser. Total genomic DNA was extracted from young leaf tissue using cetyltrimethylammonium bromide (CTAB) phenol chloroform extraction method [3]. Extracted DNA pellets were air dried and suspended in 50 μl of nuclease-free water and incubated at 37°C with DNase free RNase for 30 min. RNase was inactivated by incubating the tubes at 65°C for 10min. DNA was quantified using Nanodrop 8000 spectrophotometer (Thermo Scientific, Waltham, MA, USA) and 50 ng of extracted genomic DNA was electrophoresed on a 1% agarose gel and compared to lambda DNA dilution series (100, 80, 60, 40, 20, 10 ng) to confirm quality and quantity.

### 2.2 Genome Sequencing and Assembly

The ‘Stella’ sweet cherry genome was sequenced using multiple sequencing platforms. The data were primarily generated via Illumina sequencing platform where 76 × Illumina data were obtained from 2 × 100 standard Illumina HiSeq 2000 sequencing. Read files were obtained after initial sorting and filtering of the data via Illumina's standard data processing. Additional sequencing data were generated on the 454-sequencing platform accounting for 1.18 Gb data.

A reference-based assembly of cherry genomic Illumina and 454 data was performed using CLC Genomics assembler v 7.0 with the peach genome v 2.0 as the reference [4] and using the default parameters: length fraction = 0.5, Similarity fraction = 0.8. Additionally, a *de novo* assembly was generated based on Illumina reads using CLC Genomics v 7.0 with the default Illumina assembly parameters: Minimum contig length = 200, mismatch cost = 2, Insertion cost = 3, Deletion cost = 3, Length fraction = 0.5, Similarity fraction = 0.8. This assembly generated 96,080 contiguous sequences with an N50 of 4,130 from a total of 136,453,160 high quality reads.

## Ethics Statement

This work did not involve human subjects, animal experiments and data collected from social media platforms.

## CRediT authorship contribution statement

**Richard M. Sharpe:** Data curation, Writing – original draft. **Benjamin Killian:** Investigation. **Tyson Koepke:** Investigation. **Rishikesh Ghogare:** Data curation, Investigation. **Nnadozie Oraguzie:** Funding acquisition. **Matthew Whiting:** Funding acquisition, Supervision. **Lee A. Meisel:** Funding acquisition, Supervision. **Herman Silva:** Funding acquisition, Supervision. **Amit Dhingra:** Conceptualization, Funding acquisition, Supervision, Methodology, Resources, Writing – review & editing.

## Declaration of Competing Interest

The authors declare that they have no known competing financial interests or personal relationships which have, or could be perceived to have, influenced the work reported in this article.

## Data Availability

NCBI accession - SRR4280448, Type: 454 GS FLX Titanium (Original data) (NCBI). NCBI accession - SRR4280447, Type: Illumina HiSeq 2000 (Original data) (NCBI).

## Appendix Supplementary materials

Image, application 1

Image, application 2
