# Supplementary material for: Draft genome data of Prunus avium cv ‘Stella’
Source: Data Brief. 2022 Sep 17;45:108611. doi: 10.1016/j.dib.2022.108611 (PMC9508403; doi:10.1016/j.dib.2022.108611)
Supplement: Supplementary file 1 [file mmc1.pdf]

QIAGEN

Table of contents

1. GEX8ZDY02 (single) trimmed assembly summary report 3
1.1 Nucleotide distribution 3
1.2 Contig measurements (including scaffolded regions) 3
1.3 Contig measurements (excluding scaffolded regions) 4
1.4 Accumulated contig lengths 5
1.5 Summary statistics 5
1.6 Distribution of read length 6
1.7 Distribution of matched read length 6
1.8 Distribution of non-matched read length 7
1.9 Paired reads distance distribution 7

1. GEX8ZDY02 (single) trimmed assembly summary report

1.1 Nucleotide distribution

Nucleotide	Count	Frequency
Adenine (A)	54,958,920	31.1%
Cytosine (C)	33,088,918	18.7%
Guanine (G)	33,022,954	18.7%
Thymine (T)	54,847,192	31.1%
Any nucleotide (N)	638,103	0.4%

1.2 Contig measurements (including scaffolded regions)

N75	1,640
N50	4,601
N25	9,910
Minimum	31
Maximum	132,753
Average	1,642
Count	107,531
Total	176,556,087

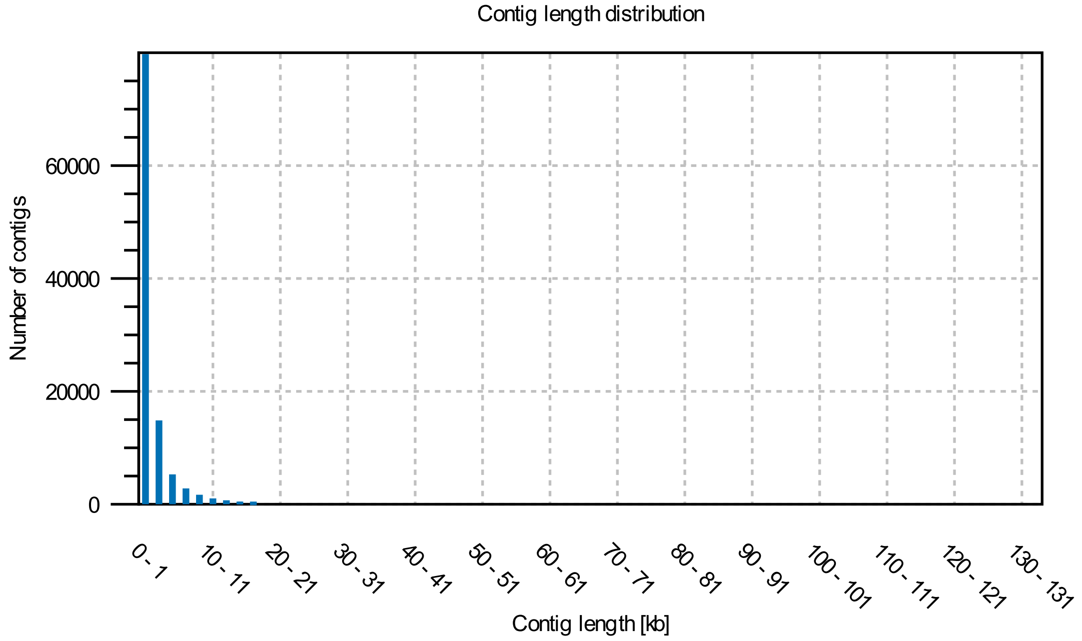

1.3 Contig measurements (excluding scaffolded regions)

N75	1,408
N50	3,926
N25	8,556
Minimum	31
Maximum	109,229
Average	1,457
Count	120,819
Total	176,067,169

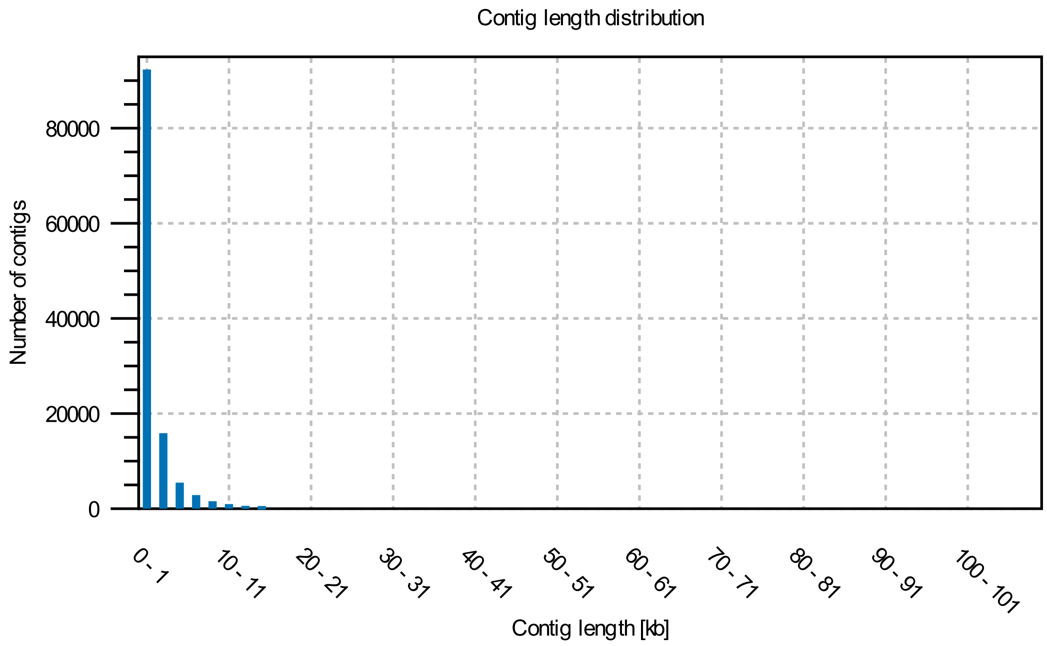

1.4 Accumulated contig lengths

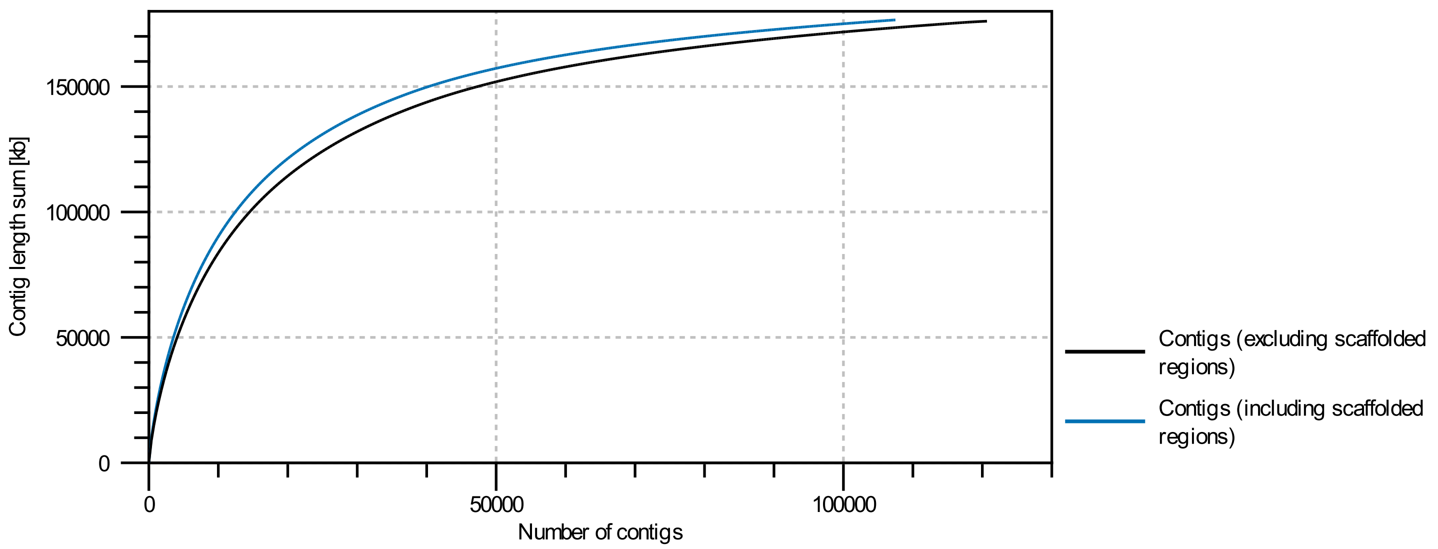

1.5 Summary statistics

	Count	Average length	Total bases
Reads	139,776,865	86.28	12,060,513,104
Matched	136,489,708	85.8	11,711,034,285
Not matched	3,287,157	106.32	349,478,819
Contigs	107,531	1,641	176,556,087
Reads in pairs	66,232,332	236.96	
Broken paired reads	52,202,355	74.38	

1.6 Distribution of read length

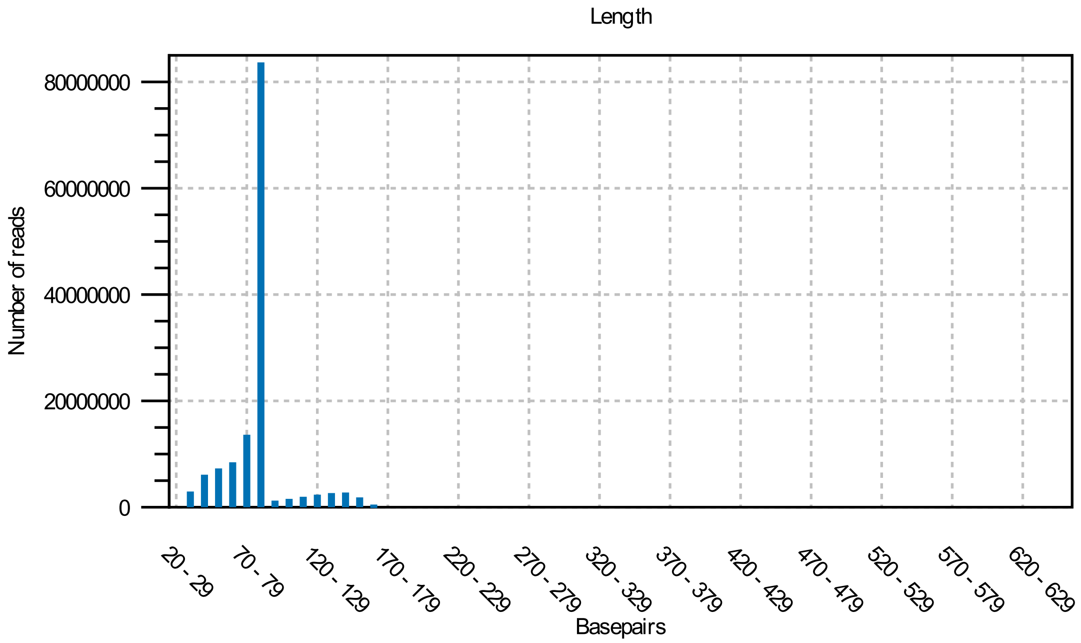

1.7 Distribution of matched read length

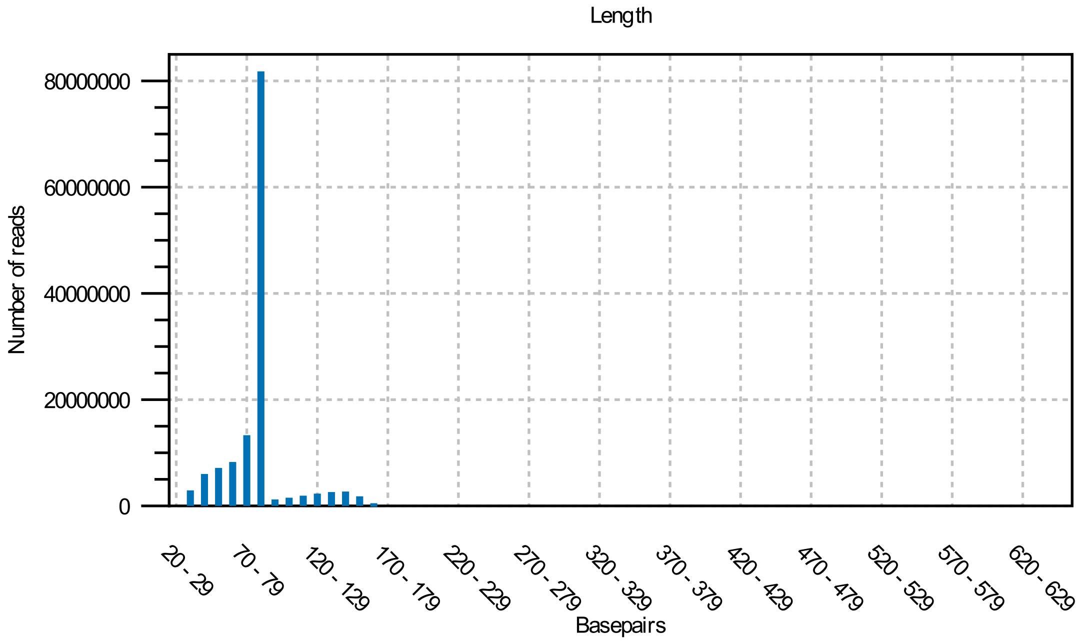

1.8 Distribution of non-matched read length

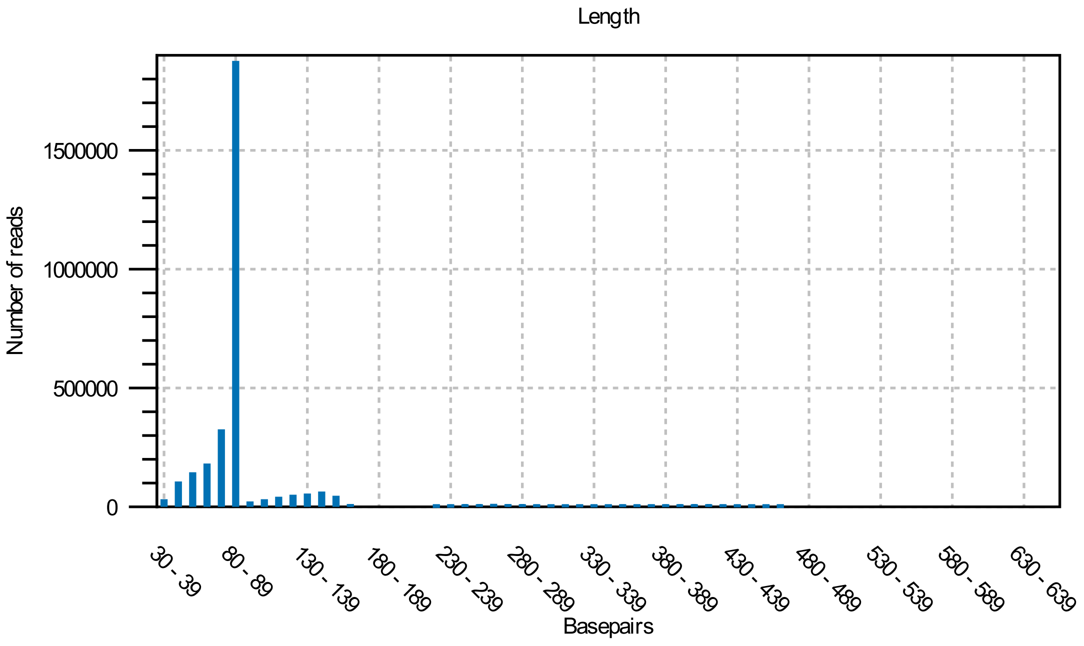

1.9 Paired reads distance distribution

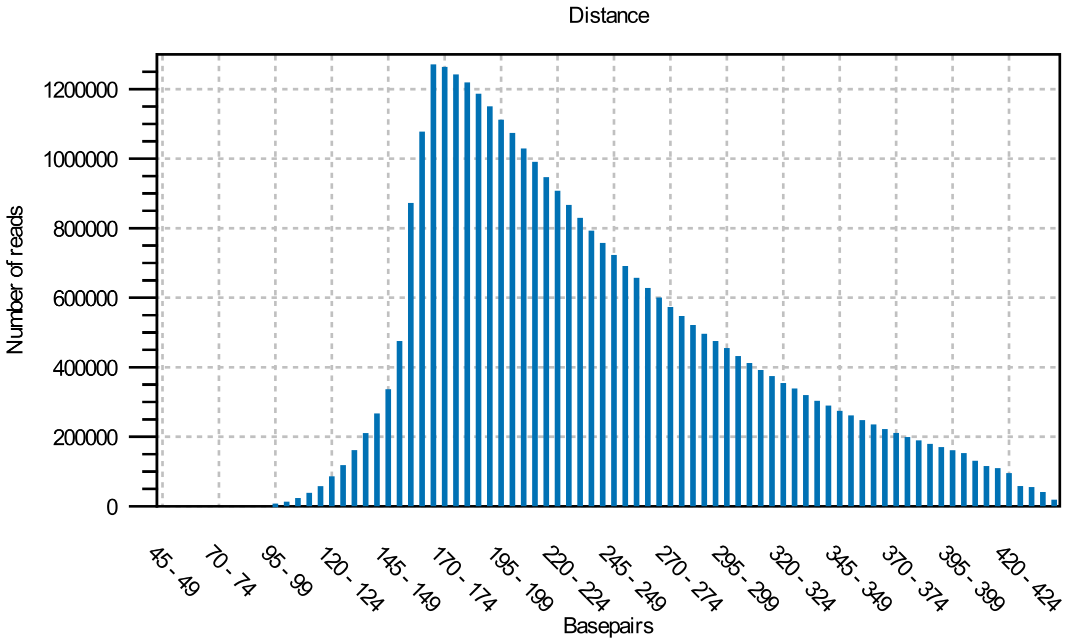

History for:

STELLA_8.15.14_assembly summary report

De Novo Assembly
15 Aug 2014 22:05:34

Version:	CLC Genomics Workbench 6.0.1
Modified by:	benjamin.kilian
Mapping mode	Map reads back to contigs (slow)
Update contigs	Yes
Automatic bubble size	Yes
Minimum contig length	200
Automatic word size	Yes
Perform scaffolding	Yes
Auto-detect paired distances	Yes
Mismatch cost	2
Insertion cost	3
Deletion cost	3
Length fraction	0.5
Similarity fraction	0.8
Create list of un-mapped reads	No
input: GEX8ZDY02 (single) trimmed	
Colorspace alignment	No
Guidance only reads	No
input: GQKO24N07 (single) trimmed	
Colorspace alignment	No
Guidance only reads	No
input: GQKO24N03 (single) trimmed	
Colorspace alignment	No
Guidance only reads	No
input: FO99VYO01 (single) trimmed	
Colorspace alignment	No
Guidance only reads	No
input: FYI7G9F01 (single) trimmed	
Colorspace alignment	No
Guidance only reads	No
input: GQKO24N05 (single) trimmed	
Colorspace alignment	No
Guidance only reads	No
input: GIND0GL02 (single) trimmed	
Colorspace alignment	No
Guidance only reads	No
input: GQKO24N06 (single) trimmed	
Colorspace alignment	No
Guidance only reads	No
input: GQKO24N04 (single) trimmed	
Colorspace alignment	No
Guidance only reads	No
input: F4EPO4W02 (single) trimmed	
Colorspace alignment	No
Guidance only reads	No

input: F37C0Q401 (single) trimmed	
Colorspace alignment	No
Guidance only reads	No
input: GQKO24N02 (single) trimmed	
Colorspace alignment	No
Guidance only reads	No
input: FP5PW3G01 (single) trimmed	
Colorspace alignment	No
Guidance only reads	No
input: F37C0Q402 (single) trimmed	
Colorspace alignment	No
Guidance only reads	No
input: FL7IZIM02 (single) trimmed	
Colorspace alignment	No
Guidance only reads	No
input: FO99VYO02 (single) trimmed	
Colorspace alignment	No
Guidance only reads	No
input: FYI7G9F02 (single) trimmed	
Colorspace alignment	No
Guidance only reads	No
input: GEX8ZDY01 (single) trimmed	
Colorspace alignment	No
Guidance only reads	No
input: Cherry_CAGATC_L003_R1_001 (paired) trimmed (paired) merged	
Colorspace alignment	No
Guidance only reads	No
input: Cherry_CAGATC_L001_R1_001 (paired) trimmed (paired) merged	
Colorspace alignment	No
Guidance only reads	No
input: Cherry_CTTGTA_L004_R1_001 (paired) trimmed (paired) merged	
Colorspace alignment	No
Guidance only reads	No
input: Cherry_CAGATC_L004_R1_001 (paired) trimmed (paired) merged	
Colorspace alignment	No
Guidance only reads	No
input: Cherry_CTTGTA_L003_R1_001 (paired) trimmed (paired) merged	
Colorspace alignment	No
Guidance only reads	No
input: Cherry_CTTGTA_L001_R1_001 (paired) trimmed (paired) merged	
Colorspace alignment	No
Guidance only reads	No
input: Cherry_CAGATC_L003_R1_001	

(paired) trimmed (paired) not merged	
Colorspace alignment	No
Guidance only reads	No
Min distance	200
Max distance	577
input: Cherry_CAGATC_L001_R1_001 (paired) trimmed (paired) not merged	
Colorspace alignment	No
Guidance only reads	No
Min distance	200
Max distance	577
input: Cherry_CTTGTA_L004_R1_001 (paired) trimmed (paired) not merged	
Colorspace alignment	No
Guidance only reads	No
Min distance	200
Max distance	577
input: Cherry_CTTGTA_L001_R1_001 (paired) trimmed (paired) not merged	
Colorspace alignment	No
Guidance only reads	No
Min distance	200
Max distance	577
input: Cherry_CAGATC_L004_R1_001 (paired) trimmed (paired) not merged	
Colorspace alignment	No
Guidance only reads	No
Min distance	200
Max distance	577
input: Cherry_CTTGTA_L003_R1_001 (paired) trimmed (paired) not merged	
Colorspace alignment	No
Guidance only reads	No
Min distance	200
Max distance	577
Comments:	3,287,157 reads were not mapped Word size: 24 Bubble size: 50 Estimated paired distance range(s): Cherry_CAGATC_L003_R1_001 (paired) trimmed (paired) not merged: 102 to 422 bp Cherry_CAGATC_L001_R1_001 (paired) trimmed (paired) not merged: 111 to 442 bp Cherry_CTTGTA_L004_R1_001 (paired) trimmed (paired) not merged: 102 to 424 bp Cherry_CTTGTA_L001_R1_001 (paired) trimmed (paired) not merged: 105 to 442 bp Cherry_CAGATC_L004_R1_001 (paired) trimmed (paired) not merged: 97 to 406 bp Cherry_CTTGTA_L003_R1_001 (paired) trimmed (paired) not merged: 108 to 436 bp

Originates from:

	GEX8ZDY02 (single) trimmed
	GQKO24N07 (single) trimmed
	GQKO24N03 (single) trimmed
	FO99VYO01 (single) trimmed
	FYI7G9F01 (single) trimmed
	GQKO24N05 (single) trimmed
	GIND0GL02 (single) trimmed
	GQKO24N06 (single) trimmed
	GQKO24N04 (single) trimmed
	F4EPO4W02 (single) trimmed
	F37C0Q401 (single) trimmed
	GQKO24N02 (single) trimmed
	FP5PW3G01 (single) trimmed
	F37C0Q402 (single) trimmed
	FL7IZIM02 (single) trimmed
	FO99VYO02 (single) trimmed
	FYI7G9F02 (single) trimmed
	GEX8ZDY01 (single) trimmed
	Cherry_CAGATC_L003_R1_001 (paired) trimmed (paired) merged
	Cherry_CAGATC_L001_R1_001 (paired) trimmed (paired) merged
	Cherry_CTTGTA_L004_R1_001 (paired) trimmed (paired) merged
	Cherry_CAGATC_L004_R1_001 (paired) trimmed (paired) merged
	Cherry_CTTGTA_L003_R1_001 (paired) trimmed (paired) merged
	Cherry_CTTGTA_L001_R1_001 (paired) trimmed (paired) merged
	Cherry_CAGATC_L003_R1_001 (paired) trimmed (paired) not merged
	Cherry_CAGATC_L001_R1_001 (paired) trimmed (paired) not merged
	Cherry_CTTGTA_L004_R1_001 (paired) trimmed (paired) not merged
	Cherry_CTTGTA_L001_R1_001 (paired) trimmed (paired) not merged
	Cherry_CAGATC_L004_R1_001 (paired) trimmed (paired) not merged
	Cherry_CTTGTA_L003_R1_001 (paired) trimmed (paired) not merged
